# *Burkholderia pseudomallei* natural competency and DNA catabolism: Identification and characterization of relevant genes from a constructed fosmid library

**DOI:** 10.1371/journal.pone.0189018

**Published:** 2017-12-18

**Authors:** Michael H. Norris, Yun Heacock-Kang, Jan Zarzycki-Siek, Andrew P. Bluhm, Ian A. McMillan, Herbert P. Schweizer, Tung T. Hoang

**Affiliations:** 1 Department of Microbiology, University of Hawaii at Manoa, Honolulu, HI, United States of America; 2 Department of Molecular Biosciences and Bioengineering, University of Hawaii at Manoa, Honolulu, HI, United States of America; 3 Department of Infectious Diseases and Pathology, University of Florida, Gainesville, Florida, United States of America; 4 Department of Molecular Genetics and Microbiology, College of Medicine and Emerging Pathogens Institute, University of Florida, Gainesville, Florida, United States of America; University of Toledo College of Medicine and Life Sciences, UNITED STATES

## Abstract

*Burkholderia* spp. are genetically and physiologically diverse. Some strains are naturally transformable and capable of DNA catabolism. *Burkholderia pseudomallei* (*Bp*) strains 1026b and K96243 and *B*. *thailandensis* strain E264 are able to utilize DNA as a sole carbon source for growth, while only strains 1026b and E264 are naturally transformable. In this study, we constructed low-copy broad-host-range fosmid library, containing *Bp* strain 1026b chromosomal DNA fragments, and employed a novel positive selection approach to identify genes responsible for DNA uptake and DNA catabolism. The library was transferred to non-competent *Bp* K96243 and *B*. *cenocepacia* (*Bc*) K56-2, harboring chromosomally-inserted *FRT*-flanked *sacB* and *pheS* counter-selection markers. The library was incubated with DNA encoding Flp recombinase, followed by counter-selection on sucrose and chlorinated phenylalanine, to select for clones that took up *flp*-DNA, transiently expressed Flp, and excised the *sacB-pheS* cassette. Putative clones that survived the counter-selection were subsequently incubated with *gfp*-DNA and bacteria were visualized via fluorescent microscopy to confirm natural competency. Fosmid sequencing identified several 1026b genes implicated in DNA uptake, which were validated using chromosomal mutants. One of the naturally competent clones selected in *Bc* K56-2 enabled *Bc*, *Bp* and *B*. *mallei* to utilize DNA as a sole carbon source, and all fosmids were used to successfully create mutations in non-naturally-competent *B*. *mallei* and *Bp* strains.

## Introduction

**Fig 1:**
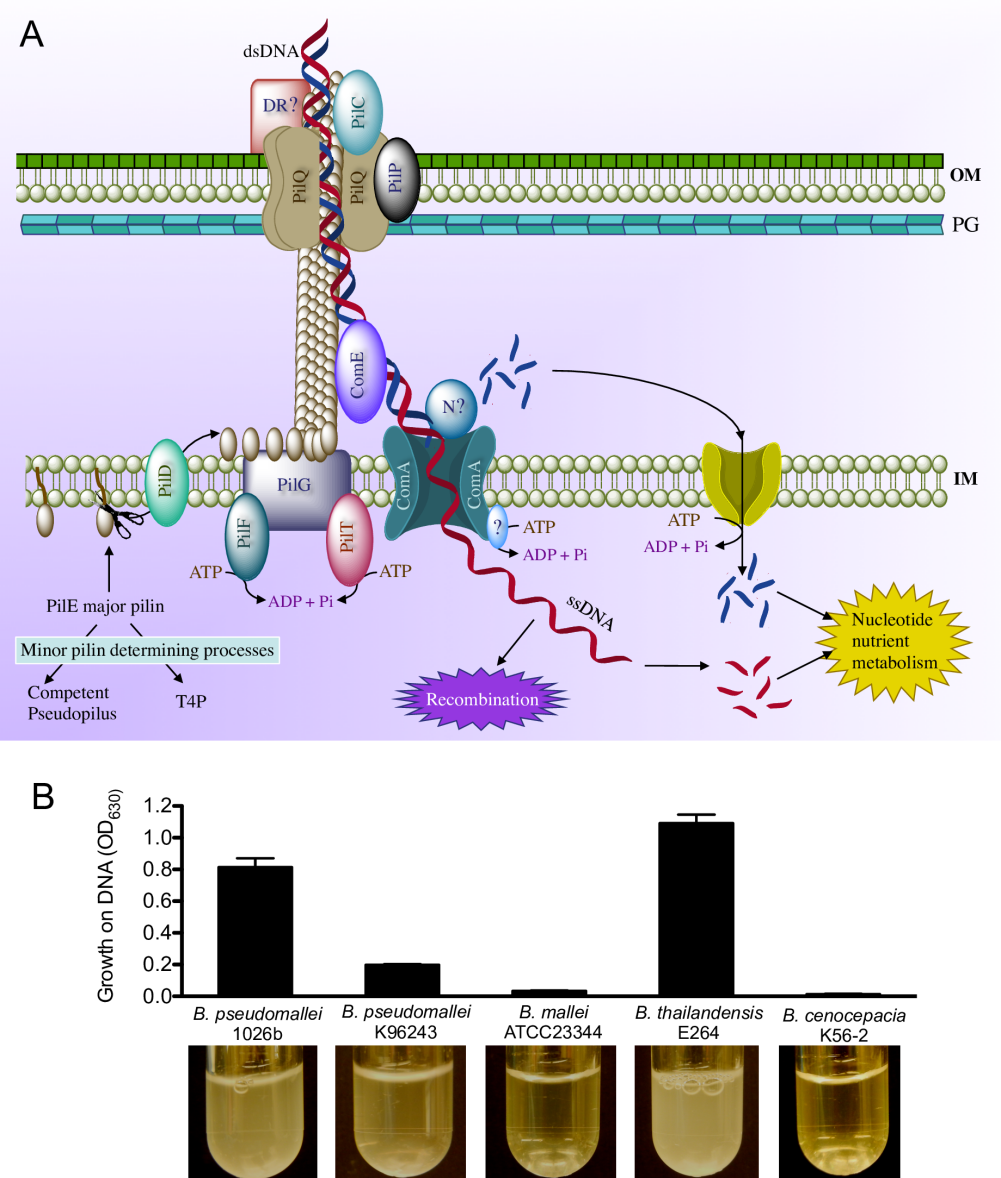
DNA uptake and utilization. (A) Model of DNA uptake in Gram-negative bacteria (see text for detail). (B) Utilization of DNA as a sole carbon and energy source in selected *Burkholderia* species. *Bp* 1026b and *B*. *thiailandensis* E264 strains exhibited heavy growth; *Bp* K96243 showed intermediate growth, while *Bc* K56-2 and *B*. *mallei* ATCC23344 were unable to grow on DNA.

Several Gram-negative bacteria are naturally competent for DNA uptake. The natural transformation system of these bacteria shares protein components common with type IV pili (T4P) and type II secretion systems (Fig 1A) [1–3], with the exception of *Helicobacter pylori* which uses components homologous to the type IV secretion system [4]. There are potentially several steps in natural transformation and DNA catabolism: competence induction, DNA binding, DNA uptake and fragmentation, and recombination and/or DNA degradation [1] (Fig 1A). It is proposed that the major pilin protein (PilE) contributes to the T4P and the natural competence pseudopilus, while the balance of these two types of pili is controlled by minor pilins [2] (Fig 1A). All pilins are processed by a PilD peptidase and assembled into the pilus structure by a PilG membrane protein and a PilF NTPase. Retraction of the pilus structure or disassembly of pilins are aided by PilG and the PilT NTPase [2]. DNA uptake initiates with the double stranded DNA (dsDNA) binding to a potential DNA receptor (DR, Fig 1A), which delivers it to the secretin made of the PilP pilot protein and 12 subunits of PilQ. PilC stabilizes the pilus structure. DNA uptake is then initiated by pseudopilus retraction. Once in the periplasm, the dsDNA is transferred by a competence protein, ComE, to the cytoplasmic membrane protein ComA. The ComA-containing translocase, with the help of an unknown ATPase [5], uses energy (ATP) to transport one strand of the dsDNA into the cytoplasm. The transportation is predicted to occur concomitantly with the degradation of the other DNA strand into nucleotides by an unknown nuclease. In support of this concomitant model of cytoplasmic ssDNA transfer and the complement strand degradation, deletion of the inner membrane channel causes dsDNA to accumulate in the periplasm [6]. Not yet demonstrated is that DNA nucleotides in the periplasm could potentially enter the cytoplasm and be used as a nutrient source. The cytoplasmic ssDNA could be used for recombination or possible degraded as nutrient. However, there could be a strong connection between natural transformation and DNA catabolism.

*Burkholderia pseudomallei* strains are genetically diverse and the etiological agents of melioidosis, a globally emerging infectious disease [7]. *B*. *pseudomallei* strains have evolved genomes of high plasticity aided by lateral and horizontal gene transfer [8, 9], which presumably occurs via conjugation, natural transformation, and transduction. Positive selection can functionally maintain novel genes and there is evidence for its contribution to *B*. *pseudomallei* pathogenesis [10]. Many environmental and clinical *B*. *pseudomallei* isolates are naturally competent for extracellular DNA uptake, which can facilitate horizontal gene transfer. Therefore, natural transformation is an avenue for direct gene transfer to competent strains and indirectly to non-competent strains, whereby naturally transformed strains with altered genomes can transfer its DNA to non-competent strains via phage or conjugation. Naturally transformable *B*. *pseudomallei* strain 1026b and *B*. *thailandensis* strain E264 can utilize DNA as sole carbon and energy source (as indicated in Fig 1B by their growth in DNA), suggesting an interconnection between natural transformation and DNA catabolism (e.g., host-cell DNA). The mechanism and various genes involved in these important processes of DNA uptake and utilization in *Burkholderia* is currently unknown.

Our long-term goal is to identify specific genes in *Bp* conferring the DNA uptake and DNA utilization phenotypes, which have the potential to be utilized for genetic manipulation in non-competent *Burkholderia*. We predicted that these phenotypes require multiple complex pathways, which could consist of genes dispersed throughout the genome. In this initial study, we took an approach to account for this complexity by screening broad genetic regions spanning the genome. We created a *Bp* 1026b fosmid clone library and tested the fosmid library for conferring functional DNA uptake (in *Bp* K96243 and *Bc* K56-2) and DNA catabolism (in *Bc* K56-2).

## Results

### Creation of a fosmid clone library

**Fig 2:**
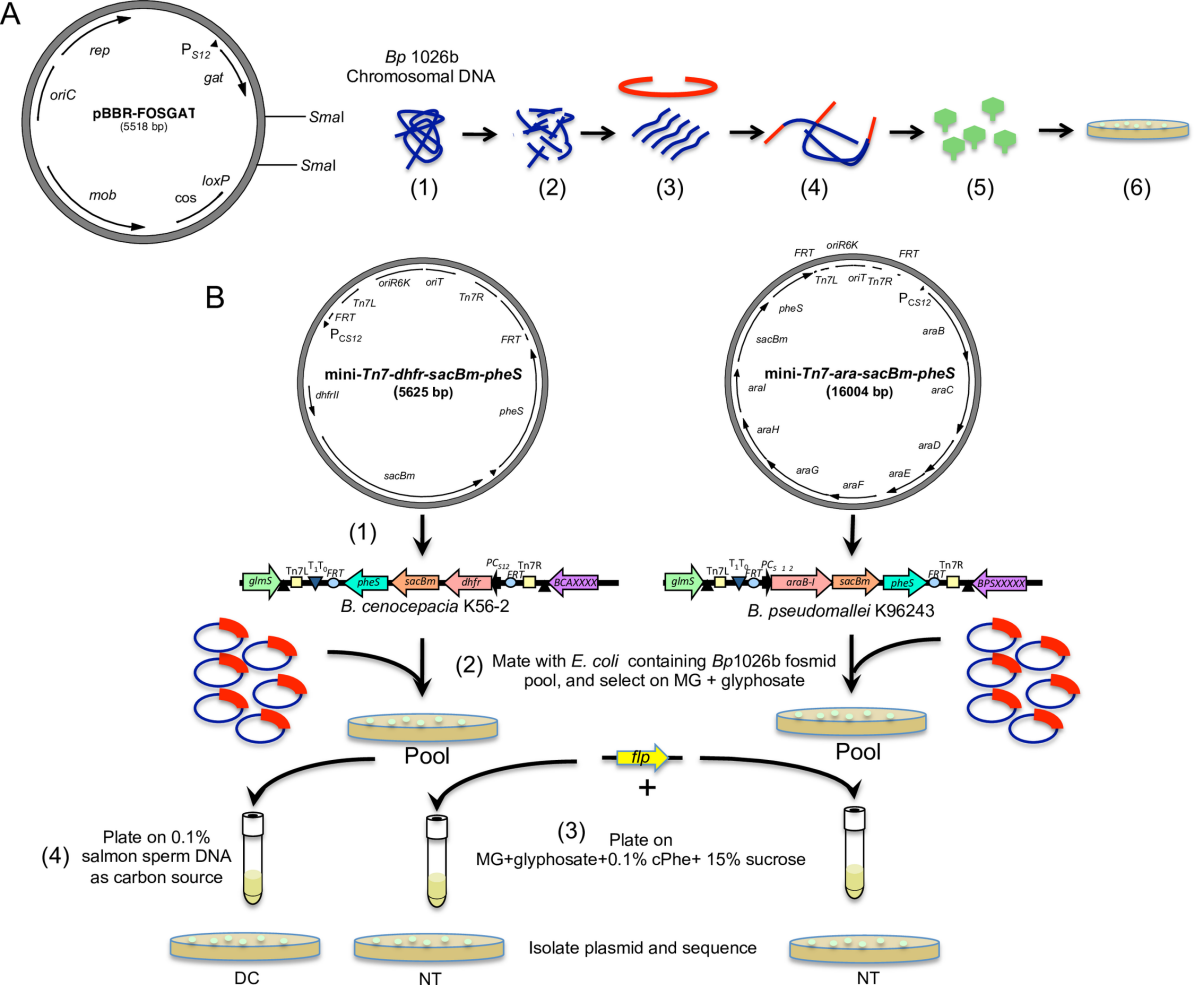
Library production and counter-selection scheme. (A) Plasmid map of an in-lab created pBBR-FOSGAT and strategy for creating the fosmid library. *Bp* 1026b chromosomal DNA (1) was sheared (2), and approximately 50-Kbp size fragments were ligated to pBBR-FOSGAT fosmid vector DNA (3 and 4; fosmid vector indicated in red). The ligated DNA was then packaged into λ-phage particles (5) and the particles are used to infect *E*. *coli* host cells (6). (B) Counter-selection scheme to screen for fosmid clones enabling DNA uptake or DNA catabolism. (1) *pheS* and *sacB* counter-selectable markers were inserted into *Bc* K56-2 and *Bp* K96243 chromosomes through minTn7 integration. (2) The *E*. *coli* library containing fosmid pool from (A) was conjugated with *Bc* K56-2 and *Bp* K96243 containing *pheS* and *sacB* counter-selectable markers. (3) Colonies from these two *Burkholderia* strains were individually pooled and incubated with *flp*-DNA, allowing transiently expressed Flp to excise the chromosomally integrated counter-selection markers and counter-selected on media containing cPhe and sucrose. (4) Alternatively, the *Bc* K56-2 pool was selected on minimal medium containing purified salmon sperm DNA as a sole carbon source.

Hypothesizing that DNA uptake and DNA catabolism for nutrients are mutually inclusive events, we set out to identify genes conferring the DNA uptake characteristic of the naturally competent *Bp* strain 1026b by screening for functional DNA uptake in *Bp* strain K96243 and *Bc* strain K56-2 or DNA catabolism in *Bc* K56-2. This was achieved by a novel experimental approach that combined the use of a low-copy broad-host-range fosmid clone library containing ~50 Kbp chromosomal DNA fragments of strain 1026b (Fig 2A) with engineered recipient strains that contained chromosomally integrated counter-selection markers, which enabled positive selection of bacteria harboring fosmid clones bestowing the ability for DNA uptake (Fig 2B). Alternatively, the *Bc* K56-2 pool was selected on minimal medium containing purified salmon sperm DNA as a sole carbon source to screen for DNA catabolism (Fig 2B). Approximately 20,000 *Escherichia coli* clones were initially created, representing more than 20-fold coverage of the *Burkholderia* species genomes. This library was mobilized into *Bp* strain K96243 and *B*. *cenocepacia* (*Bc*) strain K56-2.

### Identification of *B*. *pseudomallei* chromosomal gene fragments required for extracellular DNA uptake

Following mobilization of the fosmid clone library to non-competent *Bp* K96243 and *Bc* K56-2 containing chromosomally-inserted Flp recombinase target *(FRT)*-flanked *sacB* and *pheS* counter-selection markers, several thousand colonies were obtained for both recipient strains. After pooling the respective *Bp* and *Bc* colonies (library), the *Bc* library was plated on M9 medium containing salmon sperm DNA as the sole carbon and energy source (*Bc* K56-2 only). Additionally, fosmid clones in both species conferring the ability to uptake DNA and Flp-mediated excision of the counter-selection markers (i.e., *sacB* and *pheS* on the chromosome) were identified in two steps (Fig 2B). First, the pooled colonies were incubated with a DNA fragment encoding Flp recombinase. Once taken up into bacteria by the fosmid clones encoding the DNA uptake pathway, Flp recombinase is transiently expressed and acts on the *FRT* sites flanking the *sacB-pheS* cassette leading to excision of the counter-selectable markers from the chromosome. Second, colonies resulting from excision of the counter-selection markers from *Bp* K96243 and *Bc* K56-2 were selected by plating on sucrose and cPhe containing agar plates (Fig 2B).

Counter-selection using the entire library yielded 20–30 large and ~300 smaller colonies with *Bp* K96243 and 10–20 colonies with *Bc* K56-2. No colonies were obtained after plating of the negative controls (*Bp* K96243 and *Bc* K56-2 containing the counter-selectable markers without fosmid library). Additionally, hundreds of colonies were obtained after plating of *Bc* K56-2 on agar plates containing 0.1% salmon sperm DNA. Presumably, since wildtype *Bc* K56-2 is unable to grow on DNA as a sole carbon source (Fig 1B), these *Bc* K56-2 colonies contained fosmids that allowed growth on DNA as a sole carbon source. We next sequenced, characterized, and validated the functionality of several fosmid clones that confers DNA uptake and/or DNA catabolism.

### Several distinct *B*. *pseudomallei* genetic regions are required for DNA uptake and/or DNA catabolism

**Fig 3:**
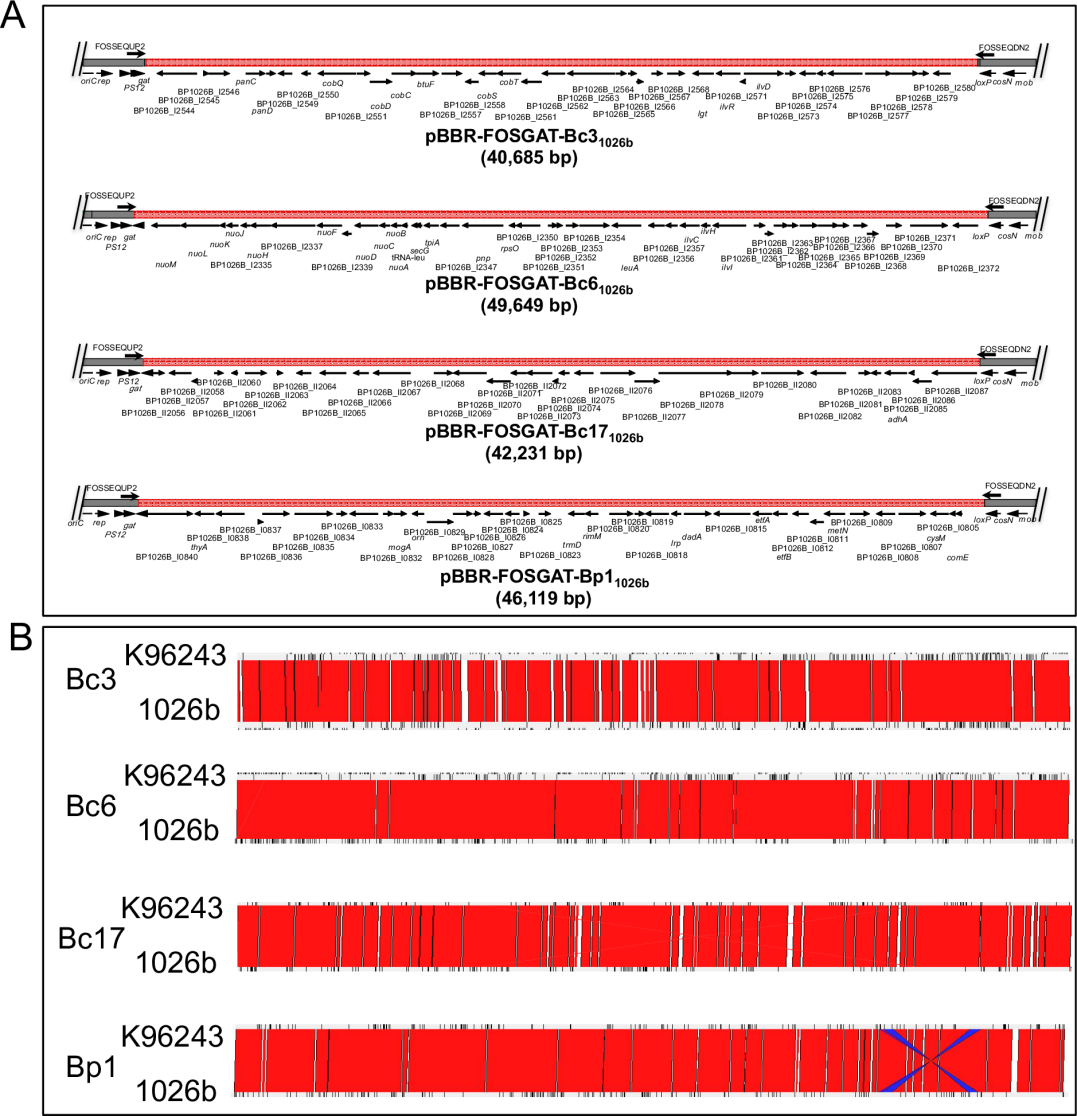
Gene content of four fosmid clones conferring natural competency and/or DNA catabolism. (A) The fosmid backbone is indicated in black and the red region depicts the extent of *Bp* 1026b genomic DNA inserted in each fosmid. Gene products with known predicted functions are indicated by gene names and those with unknown functions are labeled with gene identifications. (B) Synteny map comparing fosmid regions of *Bp* K96243 and 1026b. Red regions indicate the high level of similarity between fosmid regions in strain K96243 (non-competent) versus 1026b (competent) aligned using Artemis webACT. Identical regions are indicated in red, non-identical regions in white, and blue indicates a region that is inverted in the two strains.

We attempted to isolate fosmids clones from *Bp* and *Bc* and transform them into *E*. *coli* DH5α for identification. Between 10–20 colonies (both large and small) were processed from each of the selection method above, however, only a small number of fosmid clones were able to replicate in *E*. *coli*. Sequencing the fosmid vector-chromsomal DNA junction allowed for the identification of insert size and gene content (Fig 3A and S1 Table). Three unique inserts were identified in fosmids isolated from *Bc* (i.e., two from the counter-selection scheme, Bc3 and Bc6; and one from the selection on DNA as carbon source scheme Bc17). One unique insert was identified in fosmids isolated from *Bp* from the counter-selection scheme (Bp1; Fig 3A). The observed fosmid sizes, Bc3 ~40.1 kb, Bc6 ~49.7 kb Bc17 ~42.2 kb and Bp1 ~46.1 kb, are in the expected range based on the capacity of the phage DNA packaging capacity (~50 kb). Interestingly, the genomic regions from *Bp* 1026b in the fosmids are nearly identical to genomic regions already in the genome of *Bp* K96243, yet 1026b is naturally transformable but K96243 is not (Fig 3B).

Genes of special interest found in various fosmid clones are summarized in Table 1. At an initial glance, genes of interest on Bc3 that may be involved in DNA uptake and/or DNA catabolism include BP1026B_I2578, a predicted permease, and BP1026B_I2563-2562 that encode components of ABC transporters. Several hypothetical proteins of unknown function are also present and could be involved in conferring natural competency.

**Table 1:** Characterization of selected genes from fosmids.

Gene ID	Gene Name	Gene Function	Mutant growth defect in DNA?^*a*^	Mutant defect in *gfp* uptake?^*b*^
**Gene of interest on fosmid pBBR-FOSGAT_Bc3_1026B**				
BP1026B_I2578	Putative permease	Members of this family are predicted integral membrane proteins of unknown function. They are about 350 amino acids long and contain about 6 transmembrane regions. They are predicted to be permeases although there is no verification of this.	ND^*c*^	ND
BP1026B_I2574	Hypothetical	This family is made up of members from various *Burkholderia* spp. The function is unknown.	ND	ND
BP1026B_I2566	Hypothetical	This family of proteins has no known function.	No	No
BP1026B_I2563	Putative TonB dependent outer membrane receptor	Outer membrane receptor proteins that carry out high-affinity binding and energy-dependent uptake of specific substrates into the periplasmic space.	No	No
BP1026B_I2562	Putative transmembrane ABC transporter permease	Transmembrane component of the ABC transporter permease.	No	No
**Gene of interest on fosmid pBBR-FOSGAT_Bc6_1026B**				
BP1026B_I2371	Putative membrane protein	Probable function in catalysis of the transfer of nucleobases, nucleosides, nucleotides and nucleic acids across the membrane. Similar to *Ralstonia solanacearum* and *Neisseria meningitidis* probable transmembrane protein containing ComEC-rec2 domain.	Yes	Yes
BP1026B_I2369	Putative lysine decarboxylase	Hits by this model show a low level of similarity to and suggest an evolutionary relationship of the subfamily to the DprA/Smf family of DNA-processing proteins involved in chromosomal transformation with foreign DNA.	No	No
BP1026B_I2363	Putative transmembrane protein	This family of proteins is annotated as transmembrane proteins however this cannot be confirmed. Currently no function is known.	No	No
**Gene of interest on fosmid pBBR-FOSGAT_Bc17_1026B**				
BP1026B_II2056	Putative regulator	Crp/Fnr family transcriptional regulator	Yes	Yes
BP1026B_II2062	Hypothetical protein	44% identity at amino acid level to *B*. *melitensis* predicted lysine decarboxylase. Predicted Rossmann fold nucleotide-binding protein.	Yes	No
BP1026B_II2080	Putative signal sensor/response regulator	Somewhat similar to *Pseudomonas aeruginosa pilJ*, transmembrane, signal transduction network which controls pili extension and retraction	Yes	Yes
BP1026B_II2082	Putative protein	Putative methyl accepting transducer protein	No	No
**Gene of interest on fosmid pBBR-FOSGAT_Bp1_1026B**				
BP1026B_I0820	Hypothetical protein	Function unknown	ND	ND
BP1026B_I0819	Hypothetical protein	Membrane protein of unknown function, similar to Type IV pilus assembly protein	ND	ND
BP1026B_I0809	Hypothetical protein	Function Unknown	No	No
BP1026B_I0804	ComE	37.5% identity at nucleotide level to Ralstonia ComEA related signal protein. Helix-hairpin-helix region. 66% similar 51% identity at the amino acid level to *Chromobacterium* competence protein. The ComEA protein in bacteria is obligatory for bacterial cell competence—the process of internalizing the exogenously added DNA.	Yes	Yes

^*a*^ 1026b::Tn24 insertion mutants of these genes were grown on DNA and degrees of growth were recorded after 36 hours. Actually data were presented in S1 Fig and “Yes” indicates significant growth defect of mutant compared to wildtype (*P*<0.05 based on unpaired *t*-test).

^*b*^ 1026b::Tn24 insertion mutants of these genes were tested for *gfp-*DNA uptake. Actually data were presented in S1 Fig and “Yes” indicates significant defect in *gfp-*DNA uptake compared to wildtype (*P*<0.05 based on unpaired *t*-test).

^*c*^ ND, not determined due to strain unavailability.

Genes of interest on Bc6 include BP1026B_I2371 (encodes a protein with 13 transmembrane helices and is predicted to transport nucleobases, nucleosides, nucleotides and/or nucleic acids from one side of a membrane to another), BP1026B_I2369 (encodes a putative lysine decarboxylase; annotated as DNA-processing protein A or DprA in *Bp* K96243 which loads the recombinase RecA onto ssDNA), and BP1026B_I2363 (encodes a putative membrane protein of unknown function).

The fosmid Bc17 was selected for in *Bc* K56-2 growing on salmon sperm DNA. Genes of interest on this fosmid include BP1026B_II2056 (a transcriptional regulator that influence catabolic pathways), BP1026B_II2062 (a hypothetical protein showing 44% identity at the amino acid level to a *B*. *melitensis* predicted lysine decarboxylase modeled to the DprA family), BP1026B_II2080 (a *pilJ* homolog), and BP1026B_II2082 (encode a signal sensor/response regulator and a putative methyl accepting transducer protein).

Fosmid Bp1, counter-selected in *Bp* K96243, contains two genes of unknown function, BP1026B_I0820 and BP1026B_I0809. BP1026B_I0804 showed high identity to known competence protein, ComE, in Gram negatives. The *Bp* ComE protein has 56% homology and 38% identity at the amino acid level to the *Neisseria meningitidis* ComEA competence protein and 74% similarity and 56% identity to the *Ralstonia solanacearum* ComEA protein.

We attempted to narrow down the potential candidate genes for DNA uptake and/or DNA catabolism using several available mutants. Characterization of approximately 200 genes on the 4 fosmid clones would be overwhelming. Therefore, we selected 12 available mutant strains from the 1026b::T24 insertion mutant library to test. Mutant strains of genes BP1026B_I2371, BP1026B_I2056, BP1026B_II2080, and BP1026B_I0804 exhibited defects in both the ability to grow on DNA and uptake *gfp-*DNA, indicating their involvement in competency and DNA catabolism (Table 1 and S1 Fig). In contrast, BP1026B_II2062 mutant strain is only defective in its ability to grow on DNA, suggesting its sole involvement in DNA catabolism (Table 1 and S1 Fig). These individual genes will be of particular interest for future investigation, which is beyond the scope of this initial study. However, we further characterize these four fosmid clones here for DNA uptake and DNA catabolism capability.

### Distinct *B*. *pseudomallei* chromosomal regions play differential roles in DNA uptake and DNA catabolism

**Fig 4:**
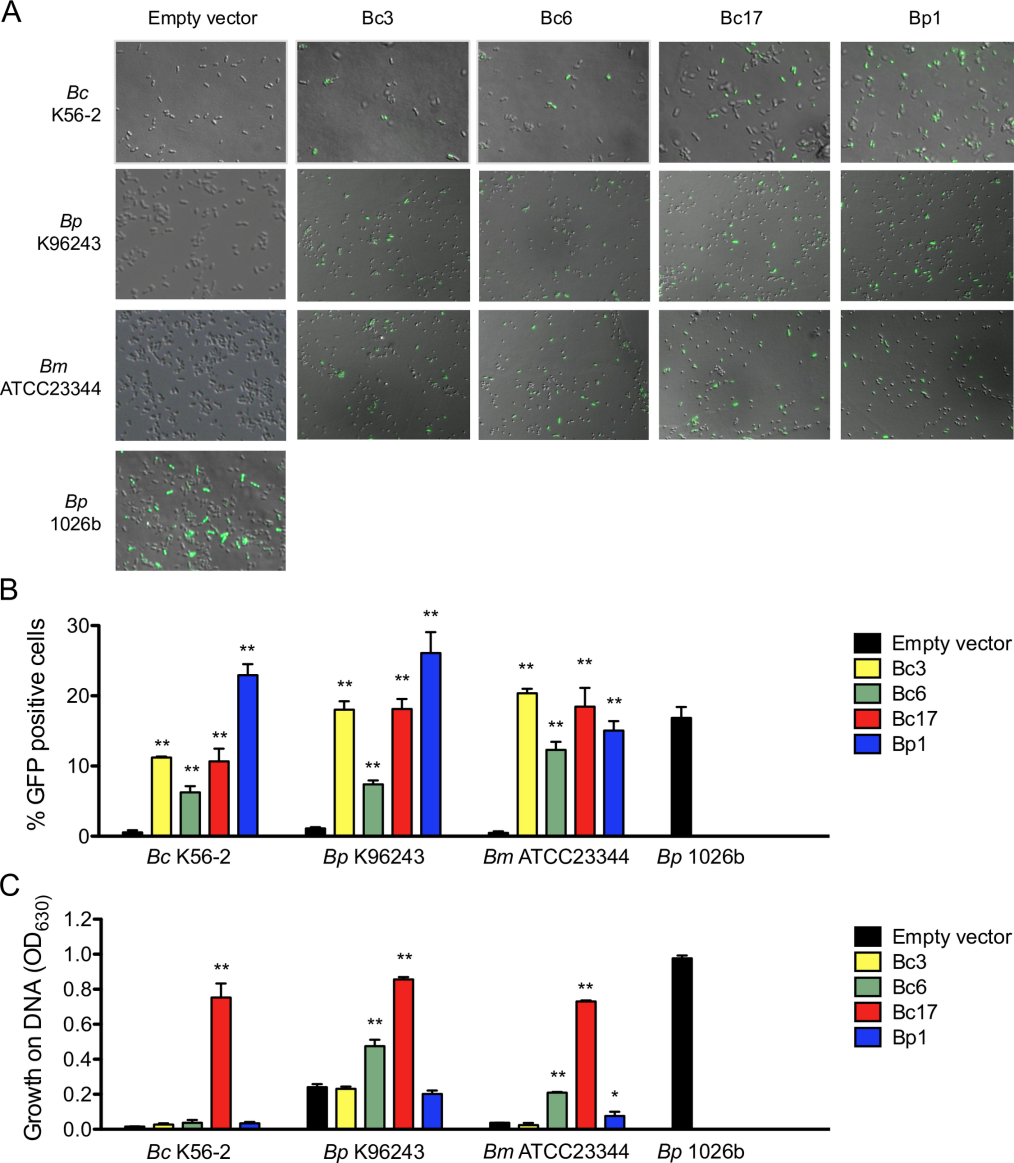
Confirmation of the ability of four fosmid clones to individually confer natural competency or growth on DNA. (A) *gfp-*DNA uptake assay to assess natural competency. Individual fluorescent bacteria are visible in the representative images (green fluorescence and DIC overlay). The percentages of cells that fluoresce following *gfp-*DNA uptake are shown in (B). Numbers are from 3 representative images from duplicate experiments. Error bars represent the SEM. Asterisks indicate the fosmid clone gave rise to significant higher %GFP positive comparing to the corresponding empty vector control in three fields (**, *P*<0.005 based on unpaired *t*-test). (C) Growth of various fosmid-containing strains in 1x M9 with 0.25% DNA done in triplicate. Error bars represent the SEM. Asterisks indicate the fosmid clone gave rise to significant higher growth in DNA comparing to the corresponding empty vector control (*, *P*<0.05 based on unpaired *t*-test; **, *P*<0.005 based on unpaired *t*-test).

Fosmids Bc3, Bc6, Bc17 and Bp1 were introduced into non-naturally competent *Burkholderia* species, *Bc* K56-2, *Bp* K96243, and *Bm* ATCC23344, along with the empty fosmid vector as a control. Following incubation with *gfp*-DNA, non-naturally competent bacteria containing the fosmids, and the empty fosmid vector were visualized under fluorescent microscopy to assess the bacterial populations that were able to uptake and express gfp-DNA. Fig 4A shows the overlay of the green channel and the DIC channel. At all levels of fluorescence excitation, there was near zero fluorescence detected above background for all the empty vector controls. As seen in the representative images, many green fluorescent bacteria populate the fosmid containing cultures. Fluorescent bacteria were counted from random images (experiments carried out in duplicate) and divided by the total number of bacteria to obtain an average percentage of fluorescent bacteria (Fig 4B). Although varied percentages of GFP-positive bacteria were observed in different species/strains, all four fosmids conferred the ability to uptake DNA in all bacteria tested.

To observe any link between DNA uptake and DNA catabolism, the same cultures used for the *gfp*-DNA incubation were grown on minimal media containing purified salmon sperm DNA as a sole carbon source. Bc17 fosmid was the only clone that enabled all three species to exhibit significant growth on DNA as a sole carbon source, indicating at least some linkage of DNA uptake to DNA catabolism (Fig 4C). Bc6 fosmid aided moderate levels of growth in *Bp* K96243 and *Bm* ATCC23344 when comparing to their empty vector controls (Fig 4C). In contrast, Bc3 fosmid failed to enable growth in any of the three species on DNA; and although significant, Bp1 fosmid only facilitated light growth of *Bm* ATCC23344 in DNA (Fig 4C).

### Exploitation of fosmid clones to genetically manipulate non-naturally competent select agent *Burkholderia* species

**Fig 5:**
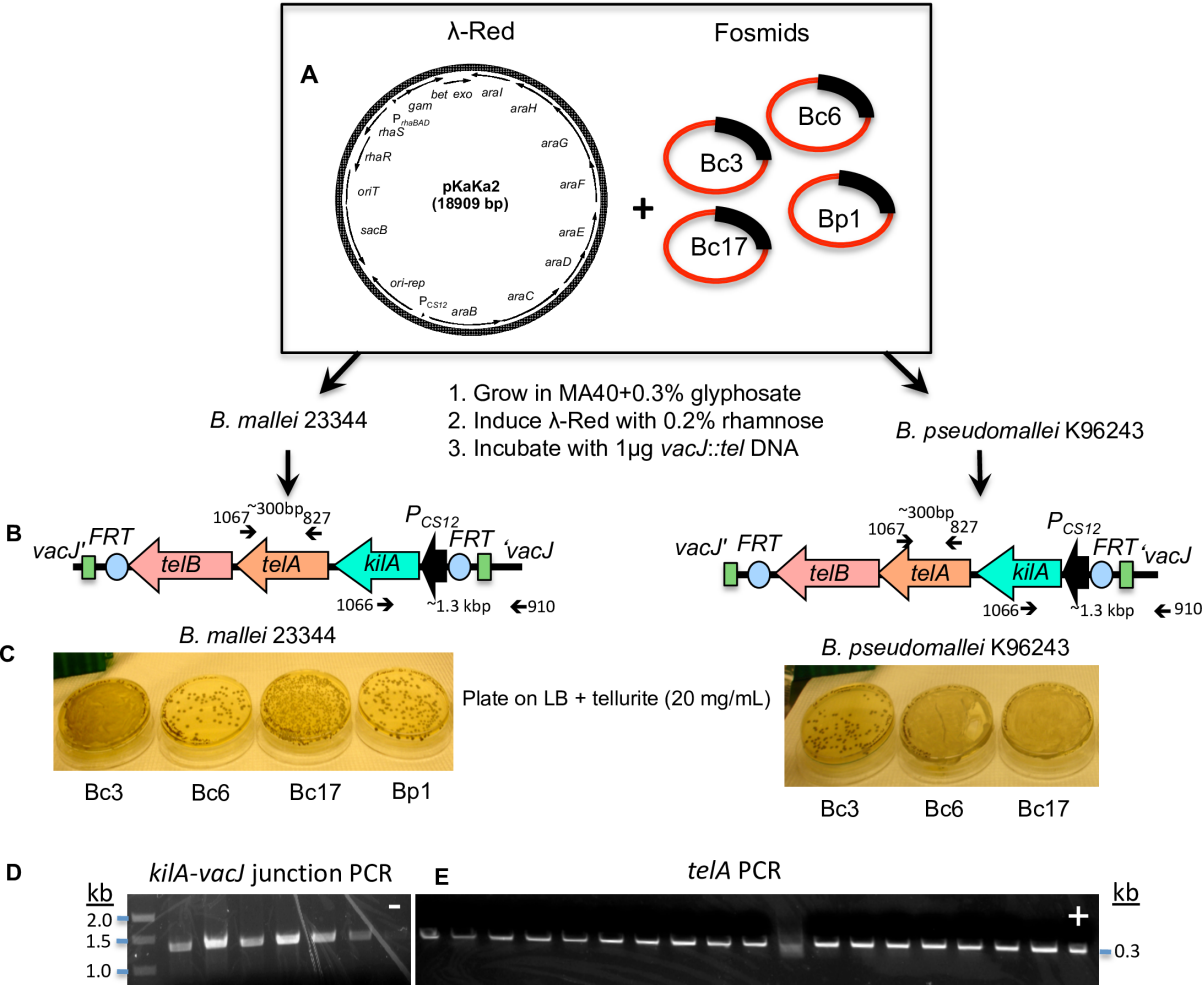
Allelic-replacement in *B*. *mallei* strain ATCC23344 and *B*. *pseudomallei* K96243 containing fosmids by natural transformation. (A) λ-Red plasmid pKaKa2 and individual fosmid clones were co-transformed into *B*. *mallei* ATCC23344 and *B*. *pseudomallei* K96243. (B) PCR products were generated containing the tellurite resistance cassette (*kilA-telA-telB*) flanked with 45-bp homologies to the *vacJ* gene. (C) Various numbers of distinct colonies were obtained. PCR was performed for the *kilA*-*vacJ* junction (D) or the *telA* gene (E) and all were positive. Two gels were shown here as representative results; “-” denotes negative control using wildtype *B*. *mallei* chromosomal DNA as template for *kilA*-*vacJ* junction PCR, and “+” denotes positive control using pwFRT-*tel* as template for *telA* PCR.

Natural transformation has previously been exploited for genetic manipulation of *B*. *pseudomallei* and *B*. *thailandensis* strains [11, 12]. We reasoned that the fosmids identified in this study could be exploited for chromosomal allelic-replacement of non-naturally competent *B*. *pseudomallei* and *B*. *mallei*. To test this notion, we assessed efficacy of λ-Red mediated mutation of the *vacJ* gene [13, 14] in the non-naturally competent *Bm* and *Bp* that harbored the fosmid candidates, using the non-antibiotic tellurite resistance marker [15] (Fig 5). *Bc* was not tested due to the lack of genetic tools for λ-Red recombination. Using *Bp* and *Bm* strains, tens to hundreds of distinct tellurite resistant colonies were obtained when expression of the λ-Red system was induced by addition of rhamnose and incubation with *vacJ’-kilA-telA-telB-‘vacJ* DNA. All fosmid clones gave rise to tranformation in both strains. All tellurite resistant colonies tested by PCR yielded the expected mutants (Fig 5E). In the absence of these fosmid clones, λ-red recombineering does not work in non-naturally competent *B*. *pseudomallei* K96243 and *B*. *mallei* ATCC23344 [16]. These data and analysis of the genomes suggested that although competency genes do exist in both strains, adding certain components may shift the balance to increase or optimize one of the several steps of the DNA uptake process to make it now more efficient (i.e., competency induction, DNA binding, DNA translocation, DNA fragmentation, DNA protection, and/or DNA recombination).

## Discussion

Natural competency is a complex process that has evolved to give some bacteria the ability to acquire new genetic material for competitive and survival advantage. Why strains within a single species are differentially competent is a poorly understood phenomenon that may have to do with regulation of the genes for DNA uptake and DNA catabolism. Recently, investigators have harnessed the bacterial ability of natural transformation to create genetic tools and methodologies that allow manipulation of the *Burkholderia* genome [12, 16]. We set out, through a gain of function screen, to identify the genes conferring natural competence within the select-agent *Burkholderiae* to lay the foundation for characterizing the biology of DNA uptake and DNA catabolism in these bacteria and to provide the potential of using competency as a tool for genetic manipulation of non-competent strains. *B*. *cenocepacia* was included in this study because it is more distantly related to *Bp* 1026b than *Bp* K96243, causing many infections in patients with cystic fibrosis and is therefore of interest to the research community. Our findings provide the framework for development of new genetic tools based on natural transformation to facilitate manipulation of the *Burkholderia* genome. Proof-of-concept studies presented here indicate that the fosmids can be exploited for genetic manipulation of non-naturally transformable species or strains.

Several novel tools were developed in the course of the present study. The broad-host-range fosmid pBBR-FOSGAT with a glyphosate non-antibiotic selection marker was successfully used to generate fosmid libraries and introduce them into diverse wild-type *Burkholderia* species. The pRK2013 based helper plasmid, pMKY, with the non-antibiotic *asd* selection marker was used to mobilize the fosmid library from *E*. *coli* to the various wild-type *Burkholderia* species. Both of these tools are welcome additions to the tool kit with non-antibiotic markers available for genetics experiments for select agent *Burkholderia* species.

Several genes encoding proteins putatively involved in competence were identified on the fosmids. All genes contained on the fosmids are present in both *Bp* 1026b and K96243 (Fig 3B), but there is a clear difference in natural transformation and DNA catabolism between these strains. This could be a result of differential gene regulation in the two strains. We hypothesize that multiple fosmid copies and/or spurious plasmid promoters could increase the levels of DNA uptake and DNA catabolism. The connection between conferring DNA uptake capability and DNA catabolism was only observed for fosmid clone Bc17. Although we have tested and identified several candidate genes that are potentially important for DNA uptake and/or DNA catabolism (Table 1), we recognize the limitations of our mutant screen to identify all genes responsible for natural competency due to the possibility of multiple homologs of each gene existing in the *Bp* genomes, and mutation in a single gene would not yield the appropriate defect. Additionally, we recognized that we only tested a limited number of genes that are available for the DNA uptake and DNA catabolism screens. A more systematic and thorough screen would require the creation and testing of hundreds of mutants based on our fosmid data, a laborious effort. Then, the significant challenge would be the future investigation into the mechanistic functions of the genes initially described in this study. However, more immediately, it would be of great interest to the *Burkholderia* community if a minimal set of genes (i.e., one or two amongst the genes in Table 1) could be identified that bestow competency in non-transformable *Burkholderia* species or strains. This would allow broader genetic manipulation amongst the *Burkholderia*.

## Materials and methods

### Strains, growth conditions, and media

All manipulations of select agent bacteria were performed in the CDC approved bio-containment facility at the University of Hawaii at Manoa, John A. Burns School of Medicine. 1026b::T24 insertion mutant library was obtained from Colorado State University and University of Washington. Derivatives of *Escherichia coli* strain EPMax10B (BioRad), E1345 and E1869 were routinely used for cloning or plasmid mobilization into *Bp* and *Bc* as described previously [15, 17]. Luria-Bertani (LB) medium (Difco) or 1x M9 minimal medium supplemented with 20 mM glucose (MG) was used to culture *E*. *coli* and *Burkholderia* strains. Selection of *gat* gene in *E*. *coli* and *Bp* strains was performed as previously described [17].

### Plasmid construction

The plasmid pCC2FOS was obtained from Epicentre in the CopyControl™ HTP Fosmid Library Production Kit. pCC2FOS was digested with *Nru*I and the *cat*-*cosN* fragment was ligated to the *Sma*I digested *rep-mob* fragment of pBBR-Gm to produce the broad-host-range plasmid pBBR-FOS. The *cat* gene was partially deleted by digestion with *Ssp*I and *Sca*I and inserting the *Eco*RV-*Xho*I digested *P*_S12_-*gat* cassette from pwFRT-*P*_S12_-*gat* [17] to produce the non-antibiotic-based broad-host-range fosmid vector pBBR-FOSGAT. Deletion of the *cat* gene, was verified by patching transformants containing pBBR-FOSGAT on LB-chloramphenicol 25 μg/ml.

The mini-Tn*7*-*ara*-*sacBm*-*pheS-FRT* vector was created by digesting the plasmid mini-Tn*7*-*bar*-*sacBm*-*pheS-FRT* with *Eco*RV and *Xho*I, blunt-ended, and ligated with the arabinose operon digested from pKaKa2 [16] and blunt-ended. The mini-Tn*7*-*dhfr-sacBm-pheS-FRT* vector was created in the same manner by sub-cloning the *Eco*RV-*Xho*I fragment containing the *dhfr* gene from pwFRT-*P*_CS12_-*dhfr* [18].

### Strain construction

Insertion of mini-Tn*7*-*ara*-*sacBm*-*pheS-FRT* into the chromosome of *Bp* strain K96243 and mini-Tn*7*-*dhfr*-*sacBm*-*pheS-FRT* into the chromosome of *Bc* strain K56-2 was carried out as previously described [17], selected on MA40 media for *ara* selection or LB + trimethoprim 100 μg/ml for *dhfr* selection, and insertions were verified by PCR. Sensitivity of both strains to 0.1% chlorinated phenylalanine (cPhe) and 15% sucrose was verified by patching.

### Fosmid library construction

Genomic DNA (gDNA) was isolated from naturally competent *Bp* 1026b using phenol-chloroform extraction. The gDNA was pipetted 100 times with a P200 pipette tip to shear the DNA. To determine proper shearing, 1 μg of sheared gDNA was analyzed on a 0.5% agarose gel with a λ DNA-Monocut Mix DNA size ladder (New England BioLabs). Five μg of the sheared gDNA was end repaired using the End-Repair Enzyme Mix according to the CopyControl™ HTP Fosmid Library Production Kit. Following end repair, the gDNA was size selected on a 0.5% agarose gel run at 30 kV overnight. Fragments approximately 35–42 kb in size were excised and eluted from the gel. The pBBR-FOSGAT was digested with *Sma*I overnight. The linearized plasmid was dephosphorylated with calf intestinal phosphatase (NEB) for 45 min and then gel purified. A 10:1 molar ratio of plasmid to sheared gDNA was mixed and ligated overnight using T4 DNA ligase (NEB). The ligation was packaged into phage heads following the CopyControl™ HTP Fosmid Library Production Kit manual. Briefly, 25 μl of MaxPlax Packaging Extracts was mixed with the ligation reaction and incubated at 30°C for 2 h. A second 25 μl aliquot of the MaxPlax Packaging Extracts was added and incubated at 30°C for another 2 h. The reaction was stopped by addition of chloroform and dilution buffer to 1 ml and stored at 4°C. Overnight culture of *E*. *coli* cloning strain E1869 was used to inoculate 50 ml of LB broth containing 10 mM MgSO_4_ and 0.2% maltose. The culture was shaken at 37°C to an OD_600_ of ~1. 100 μl of bacteria was mixed with 10 μl of the packaged phage and allowed to infect for 1 h at 37°C. The infection mixture was washed twice with 1x M9 then plated on MG + 0.3% GS + 1 mM leucine. Approximately 20,000 colonies were obtained, which represented a fosmid library with more than 20x coverage of the genome according to the following equation: *N* = ln (1-*P*) / ln (1-*f*). Where *N* is the number of colonies needed for reasonable coverage, *P* the probability of total coverage (99%), *f* the proportion of the genome contained in a single clone (40,000 kb/ 7.2 Mb). The resulting number of colonies that would give us 99% probability of obtaining a fosmid library that covers the entire genome is ~860. The ~20,000 colonies were pooled and grown in MG + 0.3% GS + 1 mM leucine. The fosmid pool was transferred from *E*. *coli* into non-competent *Bp* K96243 and *Bc* K56-2 via tri-parental mating with non-antibiotic based helper plasmid, pMKY. The plasmid pMKY is a pRK2013-based helper plasmid that has all antibiotic markers deleted (replaced by the *Pseudomonas aeruginosa* PAO1 *asd* gene) and maintained in the *E*. *coli* Δ*asd* host via *asd* complementation. The *asd* gene encodes for aspartate semialdehyde dehydrogenase, which is essential for bacterial cell wall synthesis. Library selection in *Bc* K56-2 transformants were selected on MG + 0.5% GS (v/v) while *Bp* K96243 transformants were selected on MG + 0.3% GS (v/v). Several thousand colonies were obtained from each conjugation.

### Identification of competent fosmid clones: Flp recombinase-mediated marker excision and counter-selection

*Bp* and *Bc* colonies growing on the glyphosate selection plates were pooled separately and inoculated at a 100-fold dilution into liquid MG + 0.3% GS or MG + 0.5% GS, respectively. The *flp* gene was amplified from pCD13SK-*flp*-*oriT*-*asd*_*Ec*_ [16]. The resultant ~2.6 kb fragment encoding Flp and its regulatory sequence was gel purified. After growth in MG + GS media at 37°C with shaking overnight, 2 ml of bacterial culture were concentrated 100-fold by centrifugation and incubated with 2 μg of *flp* DNA. The bacterial cell pellet and *flp* DNA were gently mixed and incubated at room temperature for 30 min. The cell-DNA mixture was then added to 3 ml of MG + GS media and allowed to recover with shaking at 37°C overnight to allow for Flp expression and excision of the chromosomally located counter-selection markers flanked by *FRT* sites. *Bc* K56-2 incubated cultures were plated on MG + 0.5% GS + 0.1% cPhe (w/v) + 15% sucrose (w/v). *Bp* K96243 and *flp* DNA incubated cultures were plated on MG + 0.3% GS + 0.1% cPhe (w/v) + 15% sucrose (w/v).

### Identification of DNA catabolism clone

*Bc* K56-2 containing the fosmid library were plated on M9 minimal agar medium containing 0.1% (w/v) purified salmon sperm DNA. Salmon sperm DNA was purchased from MPBiomedicals (Cat. #02101501; MW ~6,000,000). The salmon sperm DNA was prepared by sonicating in ten 20 sec bursts with a Kontes KT40 sonicator at maximum power using probe model ASI followed by five phenol/chloroform extractions and ethanol precipitation.

### Recovery of fosmids, sequencing and insertion determination

Fosmid DNA was isolated from *Bp* and *Bc* colonies growing on cPhe + sucrose by resuspension in 100 μl of water and heating at 100°C for 10 min to lyse all the bacteria. Cell debris was removed by centrifugation at 14,000 rpm for 5 min. Highly competent DH5α cells were prepared as previously described [18] and 40 μl aliquots of bacteria were added to 10 μl of the boil-prepped lysate in a 0.2-cm electroporation cuvette to transform the fosmids into *E*. *coli*. The recovery mixture was plated on MG + 0.3% GS + 1 mM thiamine. Colonies visible after 2 days were grown in liquid media, plasmids were isolated and the *gat* gene was verified by PCR to ensure presence of the fosmid backbone. Positive *gat* transformants were grown in 150 ml MG + 0.3% GS media for 48 h and plasmids isolated using the QIAGEN Plasmid Midi Kit according to the instructions for low copy plasmids. The oligonucleotides FOSSEQDN2 (5’- AAGTTGGGTAACGCCAGGG -3’) and FOSSEQUP2 (5’- CTGAATAAGTGATAATAAG -3’) were used to determine fosmid backbone-chromosomal DNA junction sequences. Several hundred bases of sequences were obtained for each clone and those sequences were analyzed by BLAST searches for the presence of *Bp* DNA.

### Verification of DNA uptake ability

Four selected fosmids were reintroduced into parental non-competent wild-type *Bp* and *Bc* strains by electroporation, selected on GS media, then tested for natural competency using a previously developed natural competency assay [16]. In this assay, *gfp*-DNA is amplified by PCR and 250 ng of the fragment is incubated for 30 min at room temperature with colonies from a plate (scraped from a plate after 4 days growth, adjusted to an OD_600_ of 1.5, and concentrated by centrifugation at 10,000*g*). After 45 min recovery in LB broth with shaking, the bacteria are embedded in LB + 0.5% agarose on a coverslip and observed for green fluorescence. Alternatively, select agent strains were fixed in 1% paraformaldehyde (PFA) in 1x phosphate buffered saline (PBS) for 45 min before fluorescent microscopy. Additionally, to correlate *gfp-*DNA uptake with DNA uptake and DNA use as a carbon source, the fosmid containing strains were grown in the appropriate concentration of liquid GS media overnight for fosmid maintenance. One ml of culture was then washed twice with 1x M9 to remove all carbon sources and the cell pellet resuspended in 100 μl of 1x M9. Suspended cultures were diluted 100-fold into 1x M9 media containing 0.25% (w/v) salmon sperm DNA. Growth was recorded at 72 h post-inoculation.

### λ-Red recombination in non-naturally competent select agent *Burkholderia* containing competency-providing fosmids

The λ-Red fosmid pKaKa2 was first transformed into *Bp* K96243 and *Bm* ATCC23344 with selection on MA40 media. Transformants were grown in MA40 and then each of the four competency-providing fosmids were electroporated into the strains carrying pKaKa2 and transformants were selected on MA40 + 0.3% GS. λ-Red recombineering was carried out as previously described [16]. Briefly, the tellurite resistance cassette was amplified from pw*FRT*-P_*CS12*_-*tel* [18] with oligos that incorporated 50 bp of DNA homologous to the two ends of the *Bm* and *Bp vacJ* gene (the first PCR used O-1327; 5’- **CATCTGCGGCATCGGG**CGCGGCGGCACAGTGAACGCCGCGCAAGGCGATTAAGTTGGGTA -3’ and O-1329; 5’- **GGCTGTAACCGATGTA**AGTGTCGGGTCAGTGCAGCCGGATGCTCGTATGTTGTGTGGAAT -3’. Underlined regions are regions of homology to *vacJ* and the 3’ regions are homologous to the *tel* cassette. The product was then amplified using O-1328; 5’-CATCTGCGGCATCGGG-3’ and O-1339; 5’-GGCTGTAACCGATGTA-3’ which anneal to the bolded regions indicated above in the O 1327 and O 1328 sequences to create full-length PCR products. λ-Red gene expression induced with 0.2% rhamnose (w/v) and the bacteria incubated with the tellurite selectable marker DNA flanked with 50 bp of homology to *vacJ*. One μg of DNA was then incubated with the two strains containing each of the four fosmids for a total of seven incubations and plated on LB + tellurite 20 μg/ml to select for recombinants (we did not include allelic-replacement using Bp1 in *B*. *pseudomallei* K9243, since Bp1 initially was positively selected in strain K96243). Transformants were then patched on LB + tellurite 20 μg/ml. Several colonies from each clone was verified by PCR for both the *kilA*-*vacJ* junction and the *telA* gene, using primers indicated in Fig 5B.

## Supporting information

S1 Fig Characterization of selected genes from fosmids. (A) Various 1026b::Tn24 insertional mutants were grown on DNA and degrees of growth were recorded after 36 hours. Asterisk indicates significant growth defect of mutant compared to wildtype 1026b strain (*P*<0.05 based on unpaired *t*-test). (B) 1026b::Tn24 insertion mutants were tested for *gfp-*DNA uptake. Asterisk indicates significant defect in *gfp-*DNA uptake compared to wildtype 1026b strain (*P*<0.05 based on unpaired *t*-test).
(TIF)

S1 Table All *Burkholderia* genes contained in fosmids Bc3, Bc6, Bc17, Bp1. (XLSX)
