# Supplementary figures and images for: Burkholderia pseudomallei natural competency and DNA catabolism: Identification and characterization of relevant genes from a constructed fosmid library
Source: PLoS One. 2017 Dec 18;12(12):e0189018. doi: 10.1371/journal.pone.0189018 (PMC5734746; doi:10.1371/journal.pone.0189018)

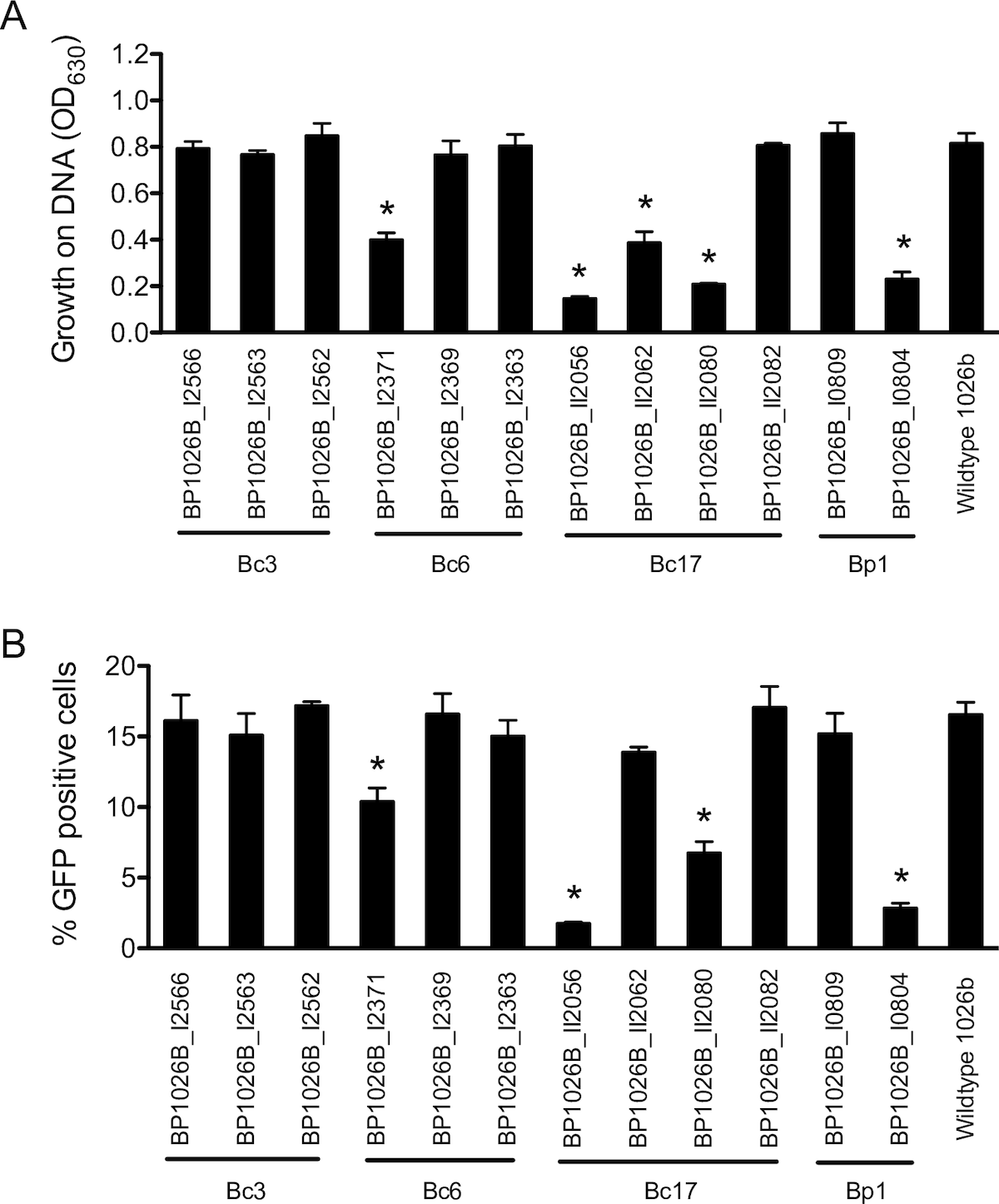

Supplement: S1 Fig — (A) Various 1026b::Tn24 insertional mutants were grown on DNA and degrees of growth were recorded after 36 hours. Asterisk indicates significant growth defect of mutant compared to wildtype 1026b strain (P<0.05 based on unpaired t-test). (B) 1026b::Tn24 insertion mutants were tested for gfp-DNA uptake. Asterisk indicates significant defect in gfp-DNA uptake compared to wildtype 1026b strain (P<0.05 based on unpaired t-test). (TIF) [file pone.0189018.s001.tif]
